# Lipoic Acid Gold Nanoparticles Functionalized with Organic Compounds as Bioactive Materials

**DOI:** 10.3390/nano7020043

**Published:** 2017-02-16

**Authors:** Ioana Turcu, Irina Zarafu, Marcela Popa, Mariana Carmen Chifiriuc, Coralia Bleotu, Daniela Culita, Corneliu Ghica, Petre Ionita

**Affiliations:** 1 Department of Organic Chemistry, Biochemistry and Catalysis, University of Bucharest, 90-92 Panduri, 050663 Bucharest, Romania; oana_turcu@yahoo.com (I.T.); zarafuirina@yahoo.fr (I.Z.); 2 Microbiology Department, Faculty of Biology, University of Bucharest, 1–3 Portocalelor Way, 060101 Bucharest, Romania; bmarcelica@yahoo.com (M.P.); carmen_balotescu@yahoo.com (M.C.C.); cbleotu@yahoo.com (C.B.); 3 Research Institute of the University of Bucharest-ICUB, 91–95 Spl. Independentei, 050095 Bucharest, Romania; 4 Romanian Academy, Ştefan S. Nicolau Institute of Virology—IVN, 285 Mihai Bravu Avenue, Sector 3, PO 77, PO Box 201, 030304 Bucharest, Romania; 5 Institute of Physical Chemistry, 202 Spl. Independentei, 060021 Bucharest, Romania; danaculita@yahoo.co.uk; 6 National Institute of Materials Physics, Laboratory of Atomic Structures and Defects in Advanced Materials, Atomistilor 105 bis, 077125 Magurele, Romania; cghica@infim.ro

**Keywords:** gold nanoparticles, lipoic acid, thioctic acid, antimicrobial, cytotoxicity, cellular cycle

## Abstract

Water soluble gold nanoparticles protected by lipoic acid were obtained and further functionalized by standard coupling reaction with 1-naphtylamine, 4-aminoantipyrine, and 4′-aminobenzo-15-crown-5 ether. Derivatives of lipoic acid with 1-naphtylamine, 4-aminoantipyrine, and 4′-aminobenzo-15-crown-5 ether were also obtained and characterized. All these were tested for their antimicrobial activity, as well as for their influence on mammalian cell viability and cellular cycle. In all cases a decreased antimicrobial activity of the obtained bioactive nanoparticles was observed as compared with the organic compounds, proving that a possible inactivation of the bioactive groups could occur during functionalization. However, both the gold nanoparticles as well as the functionalized bioactive nanosystems proved to be biocompatible at concentrations lower than 50 µg/mL, as revealed by the cellular viability and cell cycle assay, demonstrating their potential for the development of novel antimicrobial agents.

## 1. Introduction

Gold has been used for therapeutic purposes since ancient times, being mentioned in Chinese medical history in 2500 BC, still used in colloidal form in the Indian Ayurveda, and also used in western medicine for a wide range of muco-cutaneous, connective, and nervous tissues disorders [1,2].

Gold nanoparticles (Au NPs) are increasingly exploited for the design and development of novel nanomedicines, due to their ease of synthesis, characterization, and surface functionalization [3]. Different studies demonstrate their excellent antimicrobial properties, with a large spectrum including *Gram-positive* and *Gram-negative* bacteria, including multidrug resistant strains [4,5,6], as well as antiretroviral activity [7]. Functionalized gold nanoparticles have also been shown to increase the activity of antibiotics (e.g., *Vancomycin* against resistant enterococci, aminoglycosides) [8,9] or to act synergically (e.g., with *Cefaclor*, a second-generation β-lactam antibiotic, probably by targeting multiple bacterial targets, such as creating holes in the bacterial murein wall and/or interfering with the DNA condensation) [10]. However, in order to achieve the translation into clinical practice as novel tools for the antimicrobial therapy, much research is needed in order to elucidate the intimate mechanisms of action and interaction with target molecular structures, as well as the related acute and chronic toxicity issues.

Biomolecules bound on the surface of nanoparticles can simultaneously play several essential roles, like preserving the size of the main core, acting as a stabilizing ligand, and making further functionalization steps possible [11,12,13,14].

Gold nanoparticles have the advantage of versatility [15] and are commonly used for labeling with various organic compounds, and therefore can be employed as vectors containing the attached organic compounds with their desired properties. Moreover, Au NPs have unique advantages in DNA or RNA delivery to mammalian cells [16,17,18,19].

One kind of chemical reaction is based on the affinity of gold for thiol groups [9]. For example, lipoic acid (LA) is a disulfide that is well known as an antioxidant and it is reduced at intra-cellular levels into dihydrolipoic acid, a dithiol which has strong antioxidant properties [20]. In medicine it is used in many diseases, due to its antioxidant activity. Some in vivo and in vitro studies report that LA is very useful in the treatment of hyperglycemia or diabetes [21], due to its capability to stimulate the uptake of glucose into the muscle cells, where glucose is consumed.

In our study, we obtained water soluble gold nanoparticles protected by lipoic acid; these kinds of Au NPs are also known in the literature [22]. Using a standard coupling reaction of the carboxyl groups from the Au NPs with the amino groups from 4-aminoantipyrine, 4′-aminobenzo-15-crown-5 ether, and 1-naphtylamine, we obtained functionalized Au NPs and evaluated their possible biological activities, by the in vitro study of their interaction with microbial and mammalian cells.

## 2. Materials and Methods

### 2.1. Materials and Apparatus

All chemicals and solvents were purchased from Aldrich (Bucharest, Romania) or Chimopar (Bucharest, Romania). ^1^H- and ^13^C-NMR spectra were recorded on a Bruker 300 MHz instrument (Rheinstetten, Germany), using deuterated chloroform as the solvent and tetramethylsilane (TMS) as the internal standard. Infrared (IR) spectra were recorded on a Bruker FT-IR apparatus (Bremen, Germany). UV-Vis spectra were recorded in water at room temperature using an UVD-3500 double beam spectrophotometer (Labomed, LA, USA). Dinamic light scattering (DLS) analysis was performed in water on a Beckman Coulter particle size analyzer (Brea, CA, USA) using the Delsa Nano software (Beckman Coulter, Brea, CA, USA). Transmission electron microscopy (TEM) pictures were obtained using a Jeol 200 CX microscope (Jeol, Tokyo, Japan); a drop of diluted Au NPs was added on a 3 mm carbon copper grid and left to dry.

### 2.2. Synthesis of Gold Nanoparticles

LA Au NPs were obtained by dissolving 50 mg of hydrogen tetrachloroaurate into 50 mL of methanol, and under stirring a solution which was prepared by dissolving 50 mg of LA into 50 mL of methanol was added. After about 10 min, 50 mg of sodium borohydride dissolved in 5 mL of water was added to the mixture. The colour of the mixture turned from opalescent yellow-orange to black, while some hydrogen gas also evolved. After one hour, the mixture was left to settle; a black precipitate appears and this was separated by decantation of the liquid and by washing three times with methanol. For purification, the separated solid was dissolved in water and dialyzed for 24 h (diluted ammonia solution may be required in order to increase the solubility of the precipitate). The resulting purified LA Au NPs can be stored as a water solution, at pH ≈ 9 (by adding small amounts of sodium hydroxide or ammonia).

### 2.3. Functionalization of Gold Nanoparticles

As a general procedure, to 2 mL of dialyzed LA Au NPs, 20 mg of the corresponding amine (4-amino-antipyrine, 4-amino-benzo-15-crown-5 or 1-naphtylamine) was added which was dissolved before in 2 mL of isopropyl alcohol and 20 mg of 1-ethyl-3-(3-dimethyl aminopropyl)carbodiimide hydrochloride (EDC). The resulting mixture was left under stirring for three days and was then dialyzed for 1 day against 1000 mL of deionised water.

### 2.4. Synthesis of the Compounds **V**–**VII**

General procedure: To 1 mmol LA dissolved into 50 mL DCM, 1 mmol of the corresponding amine and 1.2 mmol of EDC hydrochloride was added and the mixture was stirred for three days. The solution was then extracted once with diluted aqueous hydrochloric acid (1 M) and once with diluted sodium hydrogen carbonate, and then the organic phase was dried over anhydrous sodium sulfate, filtered off, and the solvent was removed. The crude product was purified by column chromatography using silica as the stationary phase and ethyl acetate as the eluent. The yield was about 30%.

Compound **V**: ^1^H-NMR (CDCl_3_, ppm): 7.88–7.44 (m, 7H, *naphtyl*); 3.67–3.54 (t, 1H, S–C*H*); 3.18–3.10 (m, 2H, S–C*H_2_*); 2.54–2.39 (m, 2H, C*H_2_*); 2.32 (t, 2H, C*H_2_*–CO); 1.93–1.50 (m, 6H, C*H_2_*). ^13^C-NMR (CDCl_3_, ppm): 177.56; 157.09; 154.01; 128.74; 126.29; 125.89; 125.70; 121.24; 120.67; 56.39; 40.22; 38.46; 34.66; 33.81; 28.81; 24.80. IR (cm^−1^): 3320; 2927; 2850; 1625; 1531; 1448; 1242; 1087; 772; 640; 417.

Compound **VI**: ^1^H-NMR (CDCl_3_, ppm): 8.29 (s, 1H, N*H*); 7.50–7.32 (m, 5H, *phenyl*); 3.61–3.57 (m, 1H, S–C*H*); 3.56–3.12 (m, 2H, S–C*H_2_*); 3.10 (s, 3H, N–C*H_3_*); 2.49–2.45 (m, 2H, C*H_2_*); 2.34 (t, 2H, C*H_2_*–CO); 2.25 (s, 3H, C–C*H_3_*); 1.96–1.90 (m, 2H, C*H_2_*); 1.83–1.68 (m, 2H, C*H_2_*); 1.49–1.46 (m, 2H, C*H_2_*). ^13^C-NMR (CDCl_3_, ppm): 172.28; 161.76; 149.62; 134.47; 129.29; 127.10; 124.46; 108.71; 56.41; 40.20; 38.46; 36.03; 35.86; 34.66; 28.81; 25.31; 12.47. IR (cm^−1^): 3238; 3034; 2926; 2851; 1684; 1644; 1619; 1591; 1295; 759; 695; 589.

Compound **VII**: ^1^H-NMR (CDCl_3_, ppm): 8.16 (s, 1H, N*H*); 7.35 (s, 1H, C*Hphenyl*); 6.97 (d, 1H, C*Hphenyl*); 6.72 (d, 1H, C*Hphenyl*); 4.05 (s, 4H, C*H_2_*–O); 3.86–3.82 (d, 4H, C*H_2_*–O); 3.72 (s, 8H, C*H_2_*–O); 3.58–3.54 (t, 1H, S–C*H*); 3.17–3.07 (m, 2H, S–C*H_2_*); 2.45–2.38 (m, 2H, C*H_2_*); 2.40 (t, 2H, C*H_2_*–CO); 1.93–1.88 (m, 2H, C*H_2_*); 1.72–1.68 (m, 2H, C*H_2_*); 1.49–1.47 (m, 2H, C*H_2_*). ^13^C-NMR (CDCl_3_, ppm): 171.81; 148.68; 145.16; 132.33; 114.39; 112.84; 107.11; 70.95–68.34; 56.39; 53.41; 40.21; 38.45; 36.80; 34.60; 28.81; 25.41. IR (cm^−1^): 3324; 2926; 2850; 1626; 1560; 1308; 1241; 1086; 891; 640; 416.

### 2.5. Biological Activity

The antimicrobial assays were performed on the following reference strains, i.e., Gram-positive (Staphylococcus aureus ATCC 6538, Bacillus subtilis ATCC 6633, Enterococcus faecalis) and Gram-negative (Escherichia coli ATCC 8739, Pseudomonas aeruginosa ATCC 9027) bacteria and the fungus Candida albicans ATCC 10231. The quantitative assay of the antimicrobial activity was performed by the liquid medium microdilution method, in 96 multi-well plates, in order to establish the minimal inhibitory concentration (MIC). For this purpose, serial two-fold dilutions of the compounds ranging between 1000 and 1.95 µg·mL^−1^ were performed in a 200 µL volume of broth and each well was seeded with 50 µL microbial inoculum. Sterility controls (wells containing only culture medium) and culture controls (wells containing culture medium seeded with the microbial inoculum) were used. The plates were incubated for 24 h at 37 °C, and MIC values were considered as the lowest concentration of the tested compound that inhibited the visible growth of the microbial overnight cultures [23,24,25,26,27,28].

For the cellular viability assay, a HeLa cell line was seeded into 96-well plates at 5 × 10^3^ cells/well. After 24 h, binary dilutions of each compound (100, 50, and 25 µg/mL) were added and the cells were maintained for another 24 h at 37 °C, 5% CO_2_, in a humid atmosphere. The cell viability was evaluated using the CellTiter 96^®^ AQueous One Solution Cell Proliferation Assay (Promega Dexter Com S.R.L., Bucharest, Romania) measuring the absorbance at 490 nm in an enzyme-linked immunosorbent assay (ELISA) reader.

For the cell cycle assay, monolayers of HeLa cells were cultivated in RPMI 1640 (Gibco) supplemented with 10% heat-inactivated bovine serum and penicillin/streptomycin at 37 °C with 5% CO_2_. The 24 h monolayers were treated with two different concentrations (100 and 50 µg/mL) of the stock suspension of the tested nanoparticles. After that, they were maintained for another 24 h at 37 °C, in 5% CO_2_ in a humid atmosphere. After the incubation time, the cells were harvested, washed with PBS (pH 7.5), fixed in 70% cold ethanol, and maintained overnight at −20 °C. Each sample was washed in PBS, treated with 100 µg/mL RNase A (1 mg/mL), and stained with 10 µg/mL propidium iodide (PI) by incubation at 37 °C for 1 h. After PI staining of the cells, the events acquisition was performed using an Epics Beckman Coulter flow cytometer (SKU, Ramsey, MN, USA). The cytotoxicity of the tested nanoparticles was assessed by a colorimetric method using the tetrazolium salt [3-(4,5-dimethyl-2-yl)-5-(3-carboxymethoxyphenyl)-2-(4-sulfophenyl)-2H-tetrazolium; MTS] and using phenazine ethosulfate as an electron coupling reagent which forms a stable solution with MTS. The MTS tetrazolium compound is bioreduced by nicotinamide adenine dinucleotide phosphate (NADPH or NADH) dehydrogenase enzymes in metabolically active cells into a colored formazan product that is soluble in the tissue culture medium. Therefore, the value of the absorbance of the treated cells’ culture medium recorded at 490 nm is directly proportional with the percentage of viable cells. The obtained data were analyzed using FlowJo 8.8.6 software (Ashland, OR, USA).

## 3. Results and Discussion

### 3.1. Synthesis of Gold Nanoparticles

**Figure 1:**
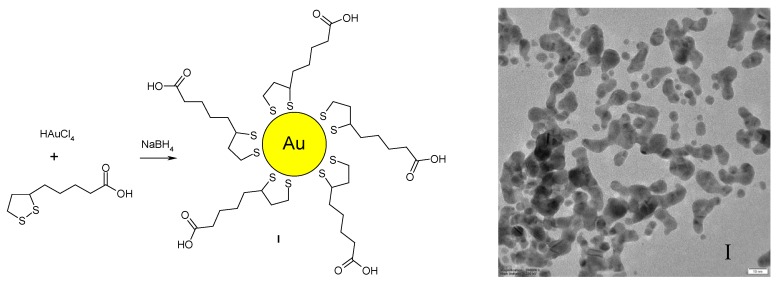
Synthesis of lipoic acid (LA) Au NPs (**left**) and their transmission electron microscopy (TEM) image (**right**). Scale bar 10 nm.

Water soluble Au NPs can be easily obtained by the reduction of gold ions with sodium borohydride in the presence of LA; while disulphide groups are attached to the gold surface, thus stabilizing the nanoparticles, the free carboxylic acid groups make possible their dispersion in water (Figure 1, left); fully coated Au NPs with an average size of 16 nm are obtained in this way (namely sample **I**), as shown by the dynamic light scattering (DLS) analysis (see Supplementary Information (SI), Figure S1). Transmission electron microscopy (TEM) analysis of the LA protected Au NPs showed an average size of about 10 nm (Figure 1, right), with a large polydispersity. It is also well known that DLS analysis give the hydrodynamic diameter of the Au NPs, not the actual diameter. Also, the ultraviolet and visible (UV-Vis) spectrum showed the expected plasmon resonance band at about 510 nm (SI, Figure S2), while the IR spectrum of the LA functionalized Au NPs showed the presence of the carboxylic groups at about 1650 cm^−1^ (a very broad band can also be noticed between 3000–3500 cm^−1^, see SI Figure S3).

### 3.2. Functionalization of Gold Nanoparticles

**Figure 2:**
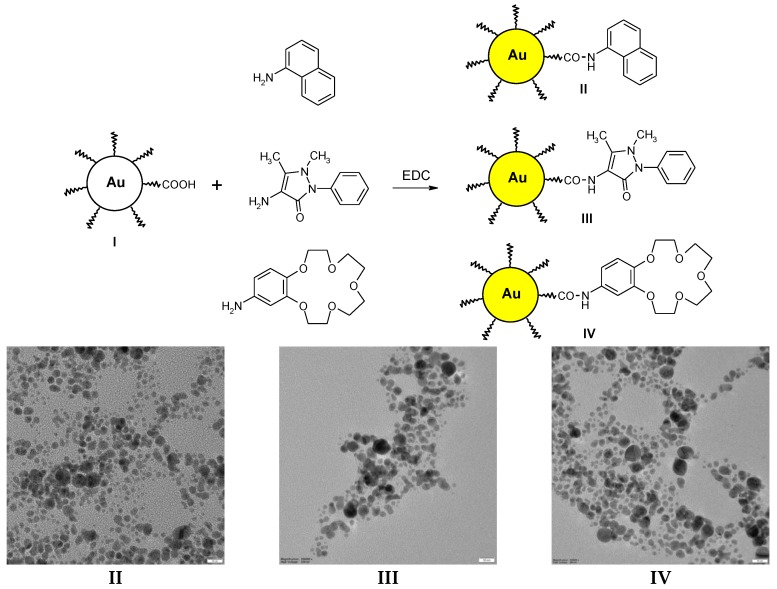
**Top**: Functionalization of Au NPs (**I**) with 1-naphtylamine, 4-aminoantipyrine, and 4′-amino-benzo-crown-5, yielding samples **II**–**IV**; **Bottom**: TEM images of samples **II**, **III**, and **IV**, respectively (scale bar 10 nm).

The functionalization of such Au NPs is easily performed using standard organic chemistry. Starting from LA Au NPs and using the coupling reaction in the presence of EDC between the COOH group from LA and the NH_2_ group from the amine (naphtylamine, amino-antipyrine, and amino-benzo-15-crown-5, Figure 2, top) it was possible to obtain Au NPs that are derivatized with the desired compounds (samples **II**–**IV**, Figure 2). These are easily purified by dialysis, as the un-reacted amines and the coupling agent pass freely through the dialysis membrane, while the Au NPs do not. These nanoparticles were also characterized by UV-Vis and IR spectroscopy. UV-Vis spectra showed that the plasmon bands were bathochromic shifted by about 10 nm, due to the functionalization of the Au NPs (SI, Figure S4).

The IR spectra of the same samples showed the presence of a strong band at 1626 cm^−1^ and a smaller band at 1685 cm^−1^, assigned to the new amidic bonds formed during the functionalization reactions (SI, Figure S5); moreover, the broad band which appeared before in the LA Au NPs between 3000–3500 cm^−1^ disappeared, while three new sharp bands emerged at 2850, 2930, and 3325 cm^−1^. These are due to the replacement of the carboxyl groups with the amide groups corresponding to the naphtylamine, amino-antipyrine, and amino-benzo-15-crown-5 derivatives. All these data confirm the functionalization of the starting LA Au NPs.

In addition, the DLS measurements of the functionalized samples showed an increase in the size of the nanoparticles (average size of 66 nm for 1-naphtylamine, 48 nm for 4-aminoantipyrine, and 86 nm for 4′-amino-benzo-crown-5, SI Figure S6a–c), meaning that some agglomeration of the Au NPs occurs during functionalization. The TEM analysis showed similar behavior as the starting LA protected Au NPs; however, the nanoparticles are better dispersed (Figure 2, bottom). Additional TEM pictures are shown in SI, Figure S7.

### 3.3. Synthesis of the Compounds **V–VII**

**Figure 3:**
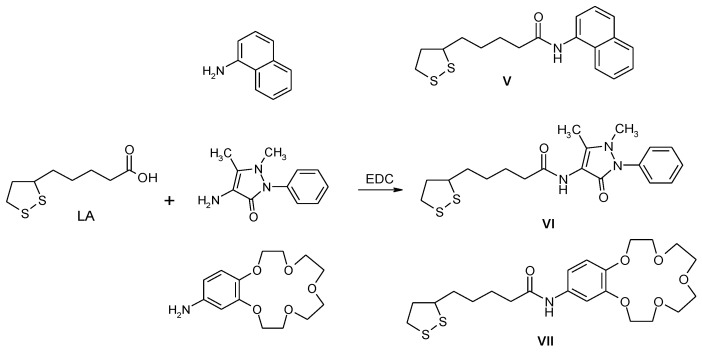
Synthesis of the compounds **V**–**VII** starting from LA.

In order to compare the biological activity of the labelled Au NPs with their organic ligand, we synthesised compounds **V**–**VII** (Figure 3) following similar coupling reactions. Although these compounds are known in the literature [29], they were characterized by ^1^H-NMR, ^13^C-NMR, and IR, thus confirming their structure (see Methods and Materials for details).

### 3.4. Biological Activity

The bare gold nanoparticles (sample **I**), as well as those functionalized with 1-naphtylamine (compound **V**), exhibited a low antimicrobial activity against all the tested microbial strains, with MIC values higher than 1 mg/mL (Table 1). Moreover, compound **V** exhibited a higher antimicrobial activity against *B. subtilis* (MIC of 0.25 µg/mL) than that exhibited by the resulting functionalized nanoparticles, demonstrating a possible inactivation of the bioactive groups during functionalization.

Compound **VI** proved to be the most active from the tested series, exhibiting a very good antimicrobial activity against *P. aeruginosa* (MIC of 0.031 mg/mL) and *B. subtilis* (MIC of 0.002 mg/mL) (Table 1). The antimicrobial activity of the functionalized nanoparticles with amino-antipyrine was also lower than that of the components, i.e., gold nanoparticles **I** and compound **VI**, probably due to the inactivation of the active groups during functionalization. The gold nanoparticles functionalized with the crown ether (sample **IV**) exhibited a very low antimicrobial activity. It is to be noticed that compound **VII** exhibited a very low MIC value against the *P. aeruginosa* strain (0.004 mg/mL), but this inhibitory effect was not observed after functionalization. The low activity can be due to the loss of the carboxyl and amino groups by functionalization.

Moreover, the very low activity noticed for samples **II**–**IV** compared with **V**–**VII** can be attributed also to the low organic content in the Au NP samples. These organic functionalized nanoparticles are composed of a 'heavy' Au metallic core protected by a 'light' single layer or thiol derivative. In the case of sample **I**, the thiol derivative is LA (Au NPs are practically protected by LA); for samples **II**–**IV**, only some LA from the Au NPs’ surface are functionalized by naphtyl, crown ether, or antipyrine moieties, as the EDC coupling reaction is not quantitative.

**Figure 4:**
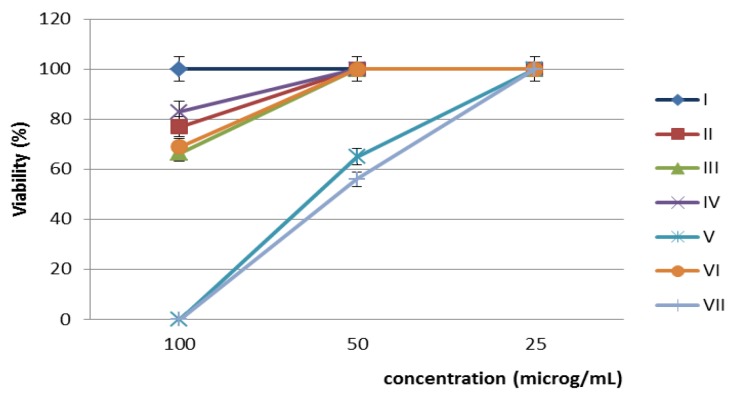
Viability (%) of HeLa cells after 24 h treatment with different concentrations of the obtained nanoparticles.

Regarding the cytotoxic assay, the tested nanoparticles exhibited different levels of cytotoxicity, depending on the type and tested concentration. Compounds **V** and **VII** proved to be the most cytotoxic at the higher tested concentrations of 100 and 50 µg/mL. The gold nanoparticles (**I**) proved to not be cytotoxic to HeLa cells, irrespective of the tested concentration. The rest of the tested nanoparticles exhibited low degrees of cytotoxicity, with this effect being observed only at the highest tested concentration of 100 µg/mL, and were not cytotoxic at the two lower tested concentrations (Figure 4).

**Figure 5:**
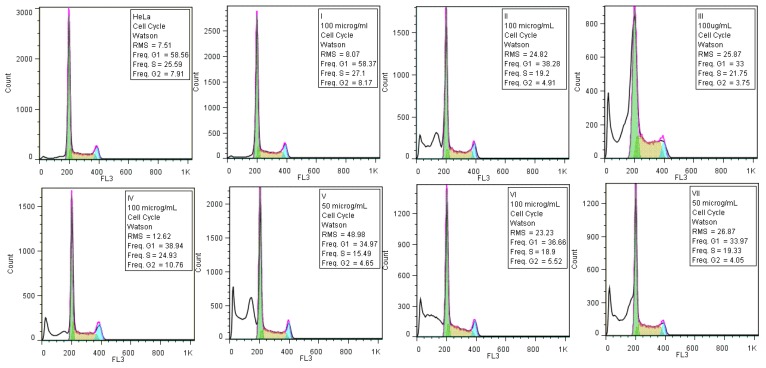
Flow cytometry diagrams of the HeLa cells’ cycle analysis grown in the presence of the tested nanoparticles.

The cellular cycle assay confirmed the results of the cellular viability assay. Thus, the gold nanoparticles (**I**) which did not affect the viability of the HeLa cells did not alter the cellular cycle phases either (Figure 4). In exchange, the other tested compounds which induced variable degrees of cytotoxicity in HeLa cells, did also alter the cellular cycle of the HeLa cells, revealed by the occurrence of a left, sub-G0 peak corresponding to cellular apoptosis, accompanied by a decrease of the percentage of cells found in the G1, S, and G2 phases (Figure 5).

## 4. Conclusions

Among the obtained nanostructures, the most effective antimicrobial properties were exhibited, in decreasing order, by compounds **VI**, **VII**, and **V** used for the functionalization of gold nanoparticles. In all cases, a decreased antimicrobial activity of the obtained bioactive nanoparticles was observed as compared to the nanosystem components, proving that a possible inactivation of the bioactive groups could occur during functionalization. However, both gold nanoparticles as well as the functionalized bioactive nanosystems proved to be biocompatible at concentrations lower than 50 µg/mL, as revealed by the cellular viability and cell cycle assay, demonstrating their potential for the development of novel antimicrobial agents.

## Figures and Tables

**Table 1:** Minimal inhibitory concentration (MIC) values of the tested compounds against the tested microbial strains.

Sample	*E. coli*	*P. aeruginosa*	*E. faecalis*	*S. aureus*	*B. subtilis*	*C. albicans*
**I**	>1	>1	>1	>1	>1	>1
**II**	>1	>1	>1	>1	1	>1
**III**	>1	>1	>1	>1	>1	>1
**IV**	>1	>1	>1	>1	>1	>1
**V**	>1	>1	>1	>1	0.25	>1
**VI**	>1	0.031	1	>1	0.002	>1
**VII**	>1	0.004	>1	>1	1	>1

## Supplementary Materials

The following are available online at http://www.mdpi.com/2079-4991/7/2/43/s1.
