# Supplementary material for: Lipoic Acid Gold Nanoparticles Functionalized with Organic Compounds as Bioactive Materials
Source: Nanomaterials (Basel). 2017 Feb 16;7(2):43. doi: 10.3390/nano7020043 (PMC5333028; doi:10.3390/nano7020043)
Supplement: Supplementary file 1 [file nanomaterials-07-00043-s001.pdf]

Supplementary Materials: Lipoic Acid Gold Nanoparticles Functionalized with Organic Compounds as Bioactive Materials

Ioana Turcu, Irina Zarafu, Marcela Popa, Mariana Carmen Chifiriuc, Coralia Bleotu, Daniela Culita, Corneliu Ghica and Petre Ionita

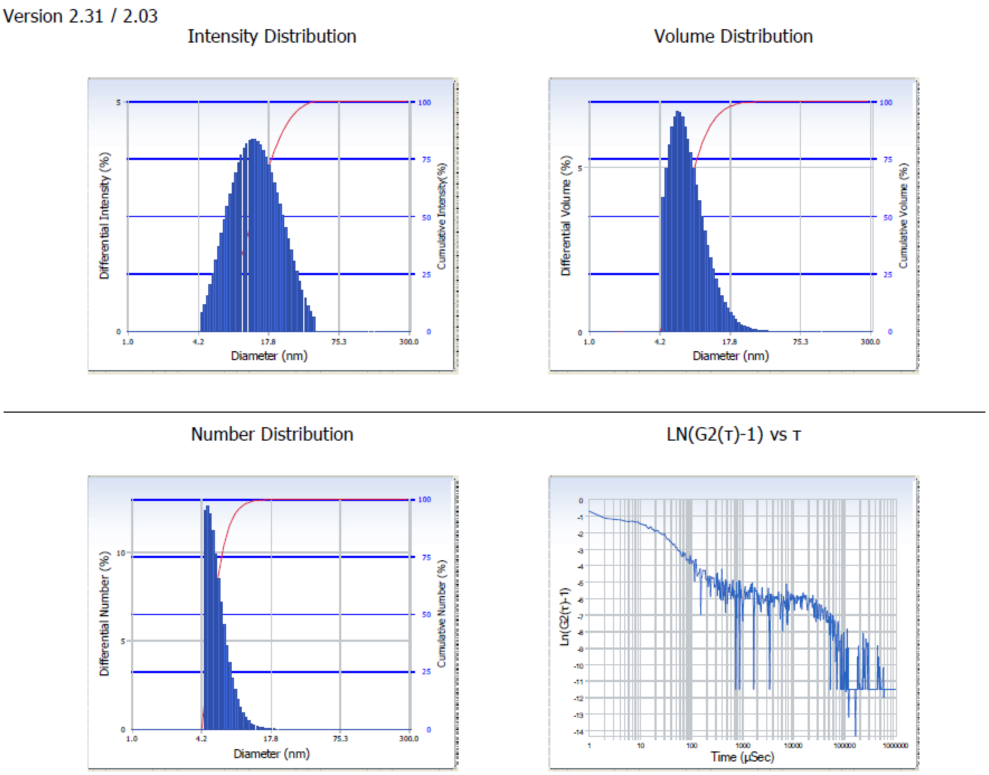

Cumulants Results			
Diameter	(d)	: 16.0	(nm)
Polydispersity Index	(P.I.)	: 0.258	
Diffusion Const.	(D)	: 3.077e-007	(cm²/sec)
Residual		: 1.709e-002	(O.K)

Measurement Condition		
Temperature	: 25.0	(°C)
Diluent Name	: WATER	
Refractive Index	: 1.3328	
Viscosity	: 0.8878	(cP)
Scattering Intensity	: 8765	(cps)

Figure S1. DLS analysis of the LA Au NPs.

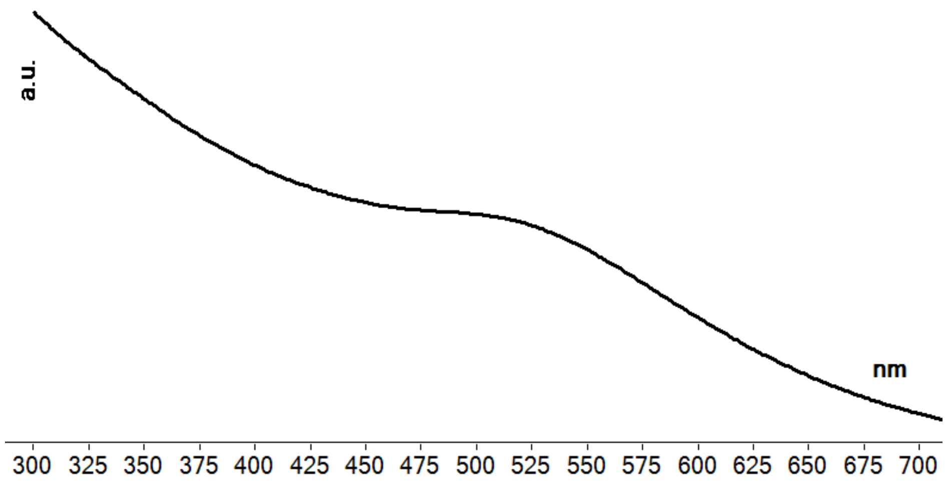

Figure S2. UV-Vis spectrum of the LA Au NPs.

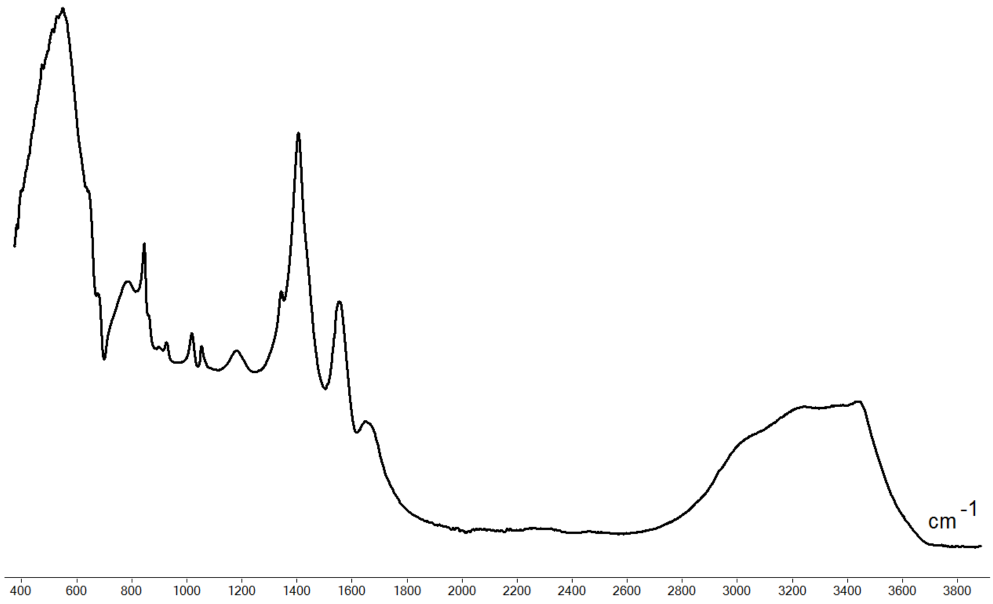

Figure S3. IR spectrum of the LA Au NPs.

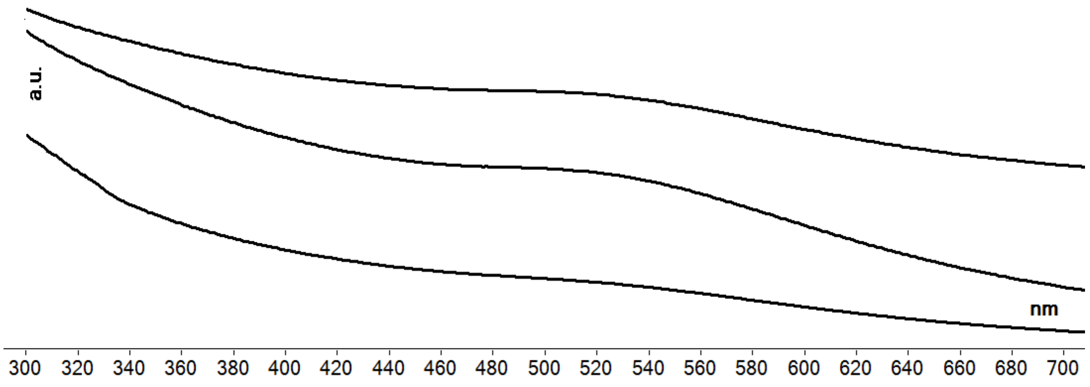

Figure S4. UV-Vis spectra of the functionalized Au NPs (from top to bottom: Au NPs functionalized with 1-naphtyl amine (**II**), 4-aminoantypirine (**III**), and 4-aminobenzo-15-crown-5 (**IV**), respectively).

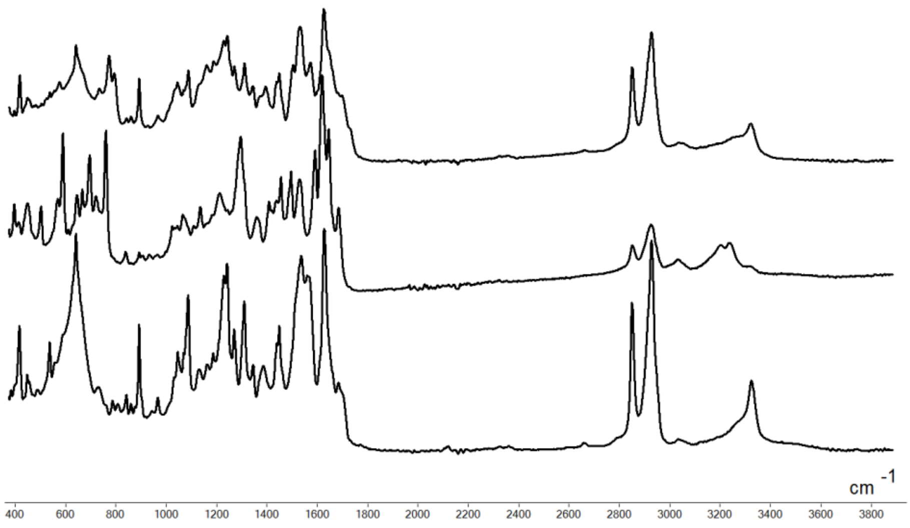

Figure S5. IR spectra of the functionalized Au NPs (from top to bottom: Au NPs functionalized with 1-naphtyl amine (**II**), 4-aminoantypirine (**III**), and 4-aminobenzo-15-crown-5 (**IV**), respectively).

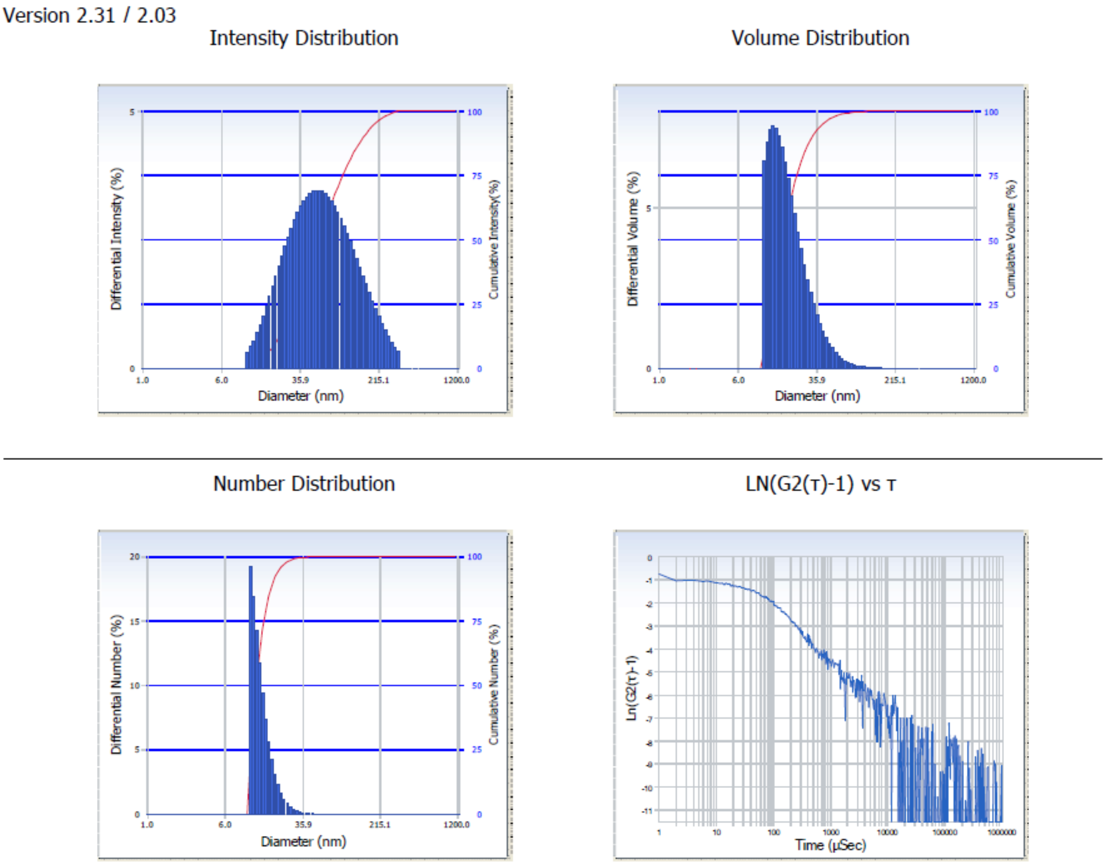

Cumulants Results				Measurement Condition			
Diameter	(d)	: 66.6	(nm)	Temperature	:	25.0	(°C)
Polydispersity Index	(P.I.)	: 0.242		Diluent Name	:	WATER	
Diffusion Const.	(D)	: 7.385e-008	(cm²/sec)	Refractive Index	:	1.3328	
Residual		: 1.568e-002	(O.K)	Viscosity	:	0.8878	(cP)
				Scattering Intensity	:	9824	(cps)

(**a**)

Cont.

Version 2.31 / 2.03

Intensity Distribution

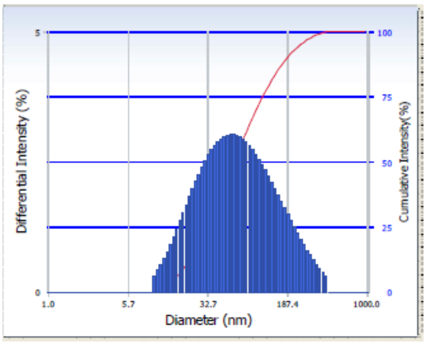

Volume Distribution

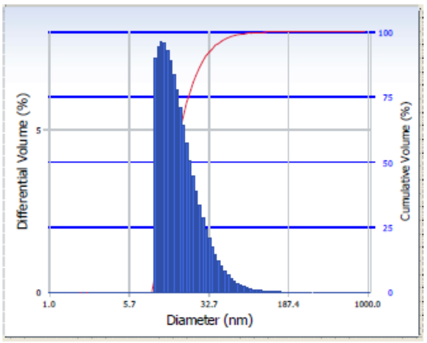

Number Distribution

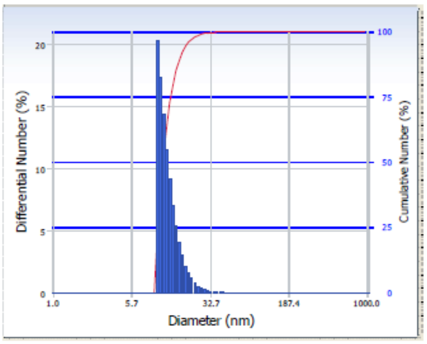

LN(G2(τ)-1) vs τ

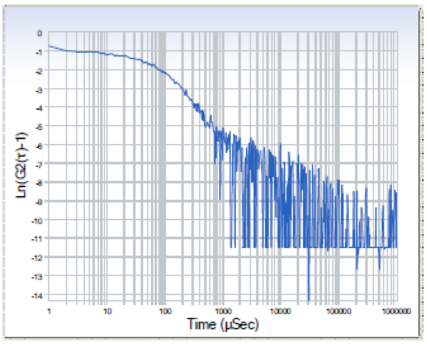

Cumulants Results			
Diameter	(d)	: 48.6	(nm)
Polydispersity Index	(P.I.)	: 0.297	
Diffusion Const.	(D)	: 1.012e-007	(cm²/sec)
Residual		: 5.960e-003	(O.K)

Measurement Condition		
Temperature	: 25.0	(°C)
Diluent Name	: WATER	
Refractive Index	: 1.3328	
Viscosity	: 0.8878	(cP)
Scattering Intensity	: 10178	(cps)

(**b**)

Cont.

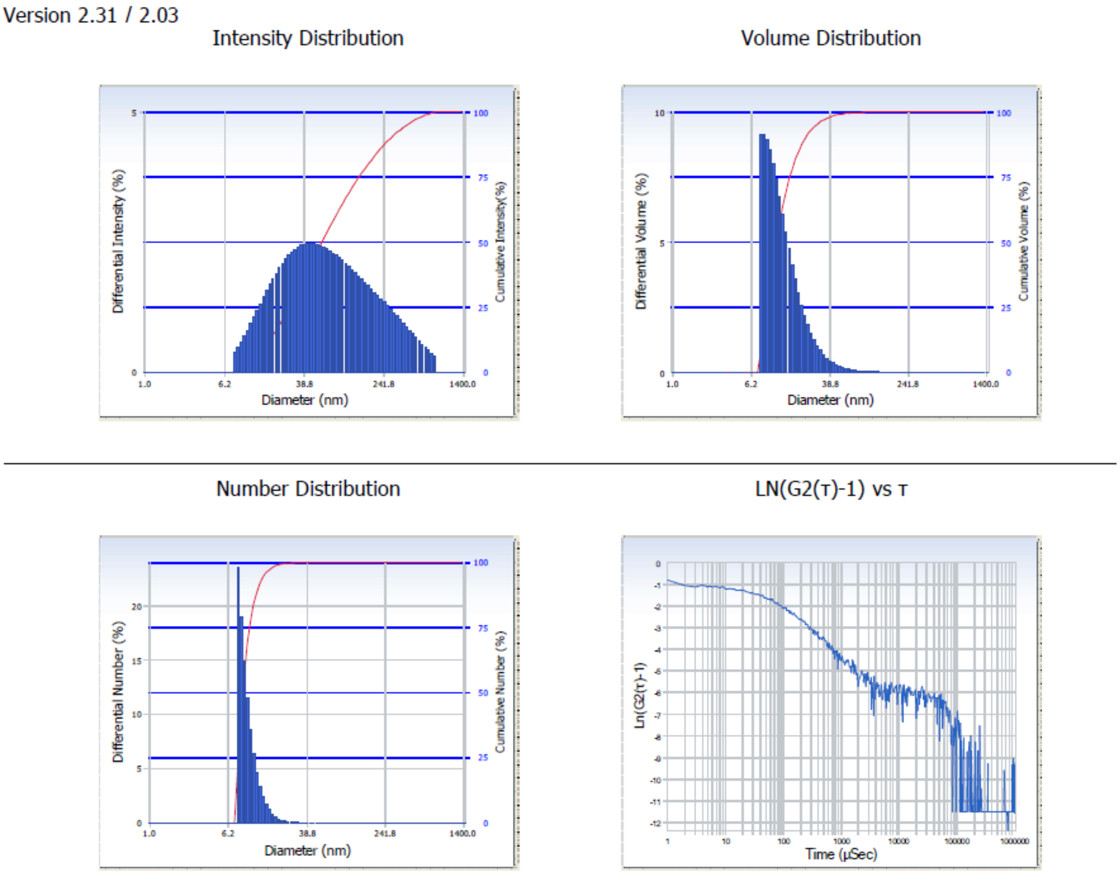

Cumulants Results			
Diameter	(d)	: 86.8	(nm)
Polydispersity Index	(P.I.)	: 0.192	
Diffusion Const.	(D)	: 5.669e-008	(cm²/sec)
Residual		: 1.202e-002	(O.K)

Measurement Condition		
Temperature	: 25.0	(°C)
Diluent Name	: WATER	
Refractive Index	: 1.3328	
Viscosity	: 0.8878	(cP)
Scattering Intensity	: 9966	(cps)

(**c**)

Figure S6. DLS analysis of the functionalized LA Au NPs with 1-naphtyl amine **(a),** 4-aminoantypirine **(b),** and 4-aminobenzo-15-crown-5 **(c)**, respectively.

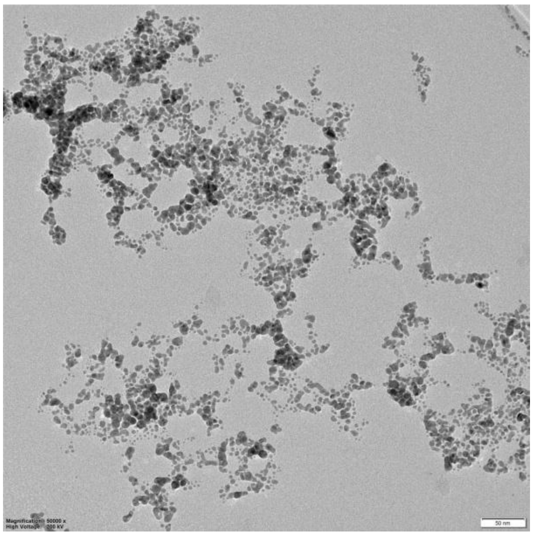

(**a**)

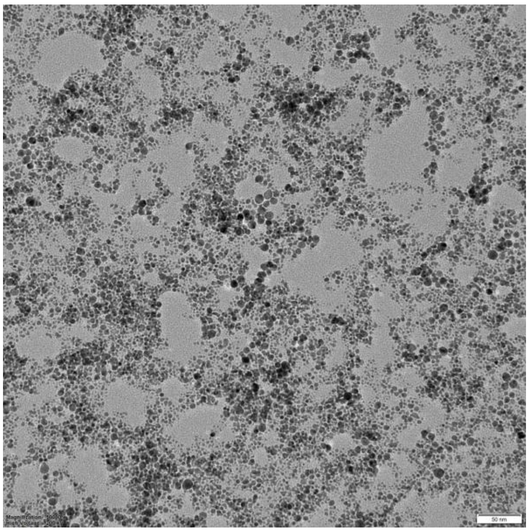

(**b**)

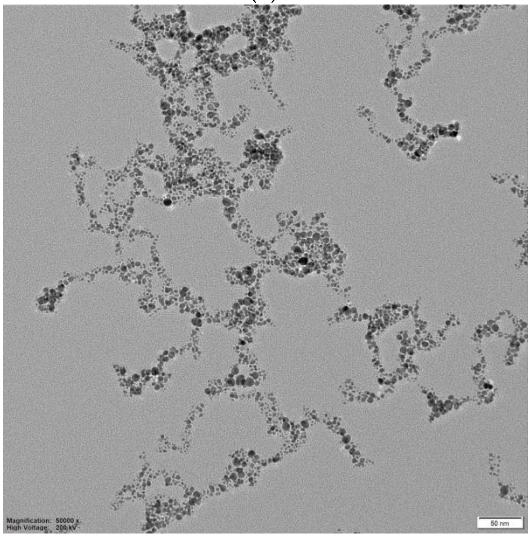

(**c**)

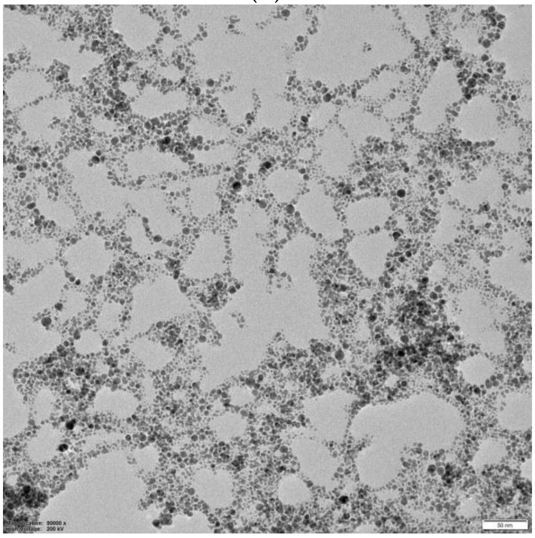

(**d**)

Figure S7. TEM analysis of the functionalized LA Au NPs (**a**), with 1-naphtyl amine (**b**), 4-aminoantypirine **(c),** and 4-aminobenzo-15-crown-5 (**d**), respectively.
